# The specific medications for pulmonary arterial hypertension at functional class III to IV: a systematic review and meta-analysis

**DOI:** 10.3389/fmed.2024.1448503

**Published:** 2024-12-12

**Authors:** Qiang Li, Hongyu Kuang, Qijian Yi, Huaan Du

**Affiliations:** ^1^Ministry of Education Key Laboratory of Child Development and Disorders, Chongqing Key Laboratory of Structural Birth Defect and Reconstruction, Department of Cardiology, National Clinical Research Center for Child Health and Disorders, Children's Hospital of Chongqing Medical University, Chongqing, China; ^2^Key Laboratory of Children's Important Organ Development and Diseases of Chongqing Municipal Health Commission, National Clinical Key Cardiovascular Specialty, Chongqing, China; ^3^Department of Cardiology, The Second Affiliated Hospital of Chongqing Medical University, Chongqing, China; ^4^Department of Cardiology, The University-Town Hospital of Chongqing Medical University, Chongqing, China

**Keywords:** pulmonary arterial hypertension, functional class III-IV, exercise capacity, cardiopulmonary hemodynamics, adverse events

## Abstract

**Purpose:**

To systematically evaluate the clinical efficacy and safety of targeted drugs in patients with pulmonary arterial hypertension (PAH) with cardiac function grades III–IV, and conduct a meta-analysis.

**Methods:**

Two researchers independently searched the PubMed, EMBASE, and Cochrane Library databases for relevant studies, with the search period extending from the establishment of the databases to March 2024. Meta-analysis was performed using statistical software Review Manager 5.4. Heterogeneity among studies was analyzed using either a random-effects model or a fixed-effects model. When the I2 value was < 50%, indicating good homogeneity, the fixed-effects model was adopted; otherwise, the random-effects model was used. For continuous variables, the 6-minute walk distance (6MWD) was expressed as the mean difference (MD), while hemodynamic parameters were represented by the standard mean difference (SMD). For categorical variables, the odds ratio (OR) was used. The confidence interval (CI) was set at 95%, and a *p* < 0.05 was considered statistically significant.

**Results:**

Ten randomized controlled trials (RCTs) involving 553 patients with PAH and cardiac function grades III-IV were ultimately included. Three RCTs targeted the endothelin pathway, five targeted the prostacyclin pathway, and two assessed the effects of combination therapy. Meta-analysis and subgroup analysis revealed that short-term monotherapy with bosentan significantly improved 6MWD by ~53.67 m (95% CI: [43.57, 63.77] meters, *p* < 0.0001) in patients with FC III-IV PAH. Additionally, prostacyclin analogs increased 6MWD by approximately 25.02 meters (95% CI: [19.22, 30.81] meters, *p* < 0.0001) in this patient population. Further hemodynamic assessments demonstrated that both bosentan monotherapy and prostacyclin analog therapy significantly reduced pulmonary vascular resistance, with SMDs of −1.07 (95% CI [-2.08, −0.06], *p* = 0.04) and −1.26 (95% CI = [−2.21, −0.32], *p* = 0.009), respectively. Analysis of the clinical efficacy of combination therapy in PAH patients revealed that while it did not significantly improve 6MWD, cardiac function improved in ~59.1% of patients (95% CI=[38.5%, 79.6%]). Safety analysis indicated that combination targeted therapy did not significantly increase the incidence of severe adverse events in PAH patients.

**Conclusion:**

Monotherapy with targeted drugs is safe and effective for patients with PAH and cardiac function grades III-IV. Combination therapy can significantly improve cardiac dysfunction in these patients without significantly increasing the risk of severe adverse events. Therefore, bosentan and prostacyclin analogs are both safe and effective options for patients with PAH and cardiac function grades III-IV. However, early combination therapy may have added clinical value in improving exercise tolerance, cardiac function, and cardiovascular remodeling in this patient population.

## Introduction

Pulmonary arterial hypertension (PAH) is a progressive disease characterized by hemodynamic features including an elevated mean pulmonary arterial pressure >20 mmHg at rest, a pulmonary artery wedge pressure ≤ 15 mmHg, and a pulmonary vascular resistance >2 Wood units (1). Epidemiological studies have revealed that the 5-year survival rate for PAH patients is < 60%. Furthermore, due to alterations in the pulmonary microcirculation, these patients often exhibit impaired exercise tolerance, hypoxemia, right heart failure, and ultimately, mortality (2, 3).

Functional class (FC) is widely used as a clinical marker in cardiovascular disease research. Though, the medical community has shifted toward risk-based stratification for treatment decisions, FC still holds regulatory significance in many countries and remains a widely reported metric in past studies. Meanwhile, FC is closely associated with survival and is thus considered an essential component of risk assessment and treatment strategies in PAH patients. And it is demonstrated that PAH patients with FC I-II typically have lower 1-year and 5-year mortality rates compared to those with FC III-IV (4).

Since the 1990s, the US Food and Drug Administration has approved targeted drugs for PAH patients who do not respond well to acute vasoreactivity testing or calcium channel blockers. These drugs target three main pathways: endothelin receptor antagonists (ERAs), phosphodiesterase type 5 inhibitors (PDE5i), prostacyclin analogs, and soluble guanylate cyclase stimulators (sGCs) (5, 6). In 2015, the ESC/ERS guidelines recommended initial combination therapy for moderate-to-high-risk PAH patients with an estimated mortality rate exceeding 5% based on the World Health Organization Functional Classification (WHO FC III-IV). This recommendation was reiterated in the 2022 ESC/ERS guidelines for high-risk patients (7, 8). However, a previous meta-analysis suggested that while combination therapy may improve exercise tolerance, functional class, and hemodynamic parameters in PAH patients, it may also be associated with more adverse events compared to monotherapy (9).

Therefore, this systematic review and meta-analysis included patients with FC III-IV PAH treated by different targeted drug strategies, in order to explore the potentially clinical efficacy and safety of targeted drug treatments in PAH patients with cardiac dysfunction. The aim of current study is to provide new insights for the clinical management of this patient population.

## Methodology

### Search strategy

The systematic review and meta-analysis were conducted adhering to the PRISMA statement (10) and Cochrane Handbook for Systematic Reviews of Interventions guidelines (11). Two independent researchers conducted searches in PubMed, EMBASE, and Cochrane Library using the terms “Pulmonary arterial hypertension OR pulmonary hypertension,” “endothelin receptor antagonists OR phosphodiesterase 5 inhibitors OR prostanoids OR soluble guanylate cyclase stimulators,” and “randomized controlled trials OR RCT OR randomized or randomly.” These terms were combined using the Boolean operator “AND”. The search was limited to studies published from the inception of each database (PubMed: 1950, EMBASE: 1974, and Cochrane Library: 1993) to March 2024. Manual searches were also conducted to complement the electronic searches.

### Inclusion and exclusion criteria

Inclusion criteria included: (1) randomized controlled studies; (2) adult patients with pulmonary arterial hypertension diagnosed as FC III or IV according to the FC evaluation criteria set by the WHO or NYHA; (3) studies involving PAH-targeted drug intervention trials; and (4) outcomes such as exercise tolerance (6MWD, cardiac function classification), hemodynamic parameters, drug adverse effects, and mortality. Exclusion criteria encompassed: (1) case reports, reviews, meta-analyses, animal studies, conference proceedings, etc.; (2) studies with uncertain or FC I-II graded patients; (3) a study focusing on pregnant populations with PH; (4) duplicate or incomplete literature.

### Literature screening and data extraction

Two authors initially screened the literature based on titles, abstracts, and results. A second screening was conducted after reading the full texts, and studies were selected based on the established criteria. Any conflicts were resolved through discussions with the corresponding author. Using a standardized extraction form, the two authors independently extracted data from the included studies, including basic information (first author's name, year of publication), intervention details (drug, type, sample size), comparator (sample size), PAH etiology, baseline 6MWD, baseline hemodynamic variables (mPAP, PVR, PCWP, CI, etc.), baseline cardiac function, and outcomes (mortality, clinical worsening events and adverse effects, 6MWD, changes in cardiac function, mPAP, PVR, CI). For the purposes of this study, clinical worsening events were defined as a composite endpoint including death, heart or lung transplantation, hospitalization due to PAH, deterioration, or lack of clinical improvement leading to discontinuation of the drug or the need for another treatment. In cases of duplicate studies, only the report with the most comprehensive and complete data was included. If PVR values in the included studies were reported in dyn/sec/cm^5^, they were converted to Wood units by dividing by 80 (12).

### Quality assessment of literature

Two researchers independently assessed the risk of bias in each study using the Cochrane risk of bias assessment tool. The quality evaluation primarily focused on six aspects: (1) random sequence generation; (2) allocation concealment; (3) blinding (participants and outcomes); (4) incomplete outcome data; (5) selective reporting; and (6) other biases.

### Statistical analysis

Meta-analysis was performed using statistical software such as Review Manager 5.4 and OpenMeta(analyst). Heterogeneity among studies was evaluated using the I^2^ statistic, where I^2^ > 50% indicated significant heterogeneity, necessitating the use of a random-effects model. Conversely, an I^2^ value of < 50% indicated good homogeneity, justifying the use of a fixed-effects model. Continuous variables, such as the 6-min walk distance (6MWD), were expressed as mean differences (MD), while hemodynamic parameters were represented by standard mean differences (SMD). Non-continuous variables were expressed as odds ratios (OR). Confidence intervals (CI) were set at 95%, and a *p* < 0.05 was considered statistically significant (13, 14).

## Results

### Basic characteristics

**Figure 1:**
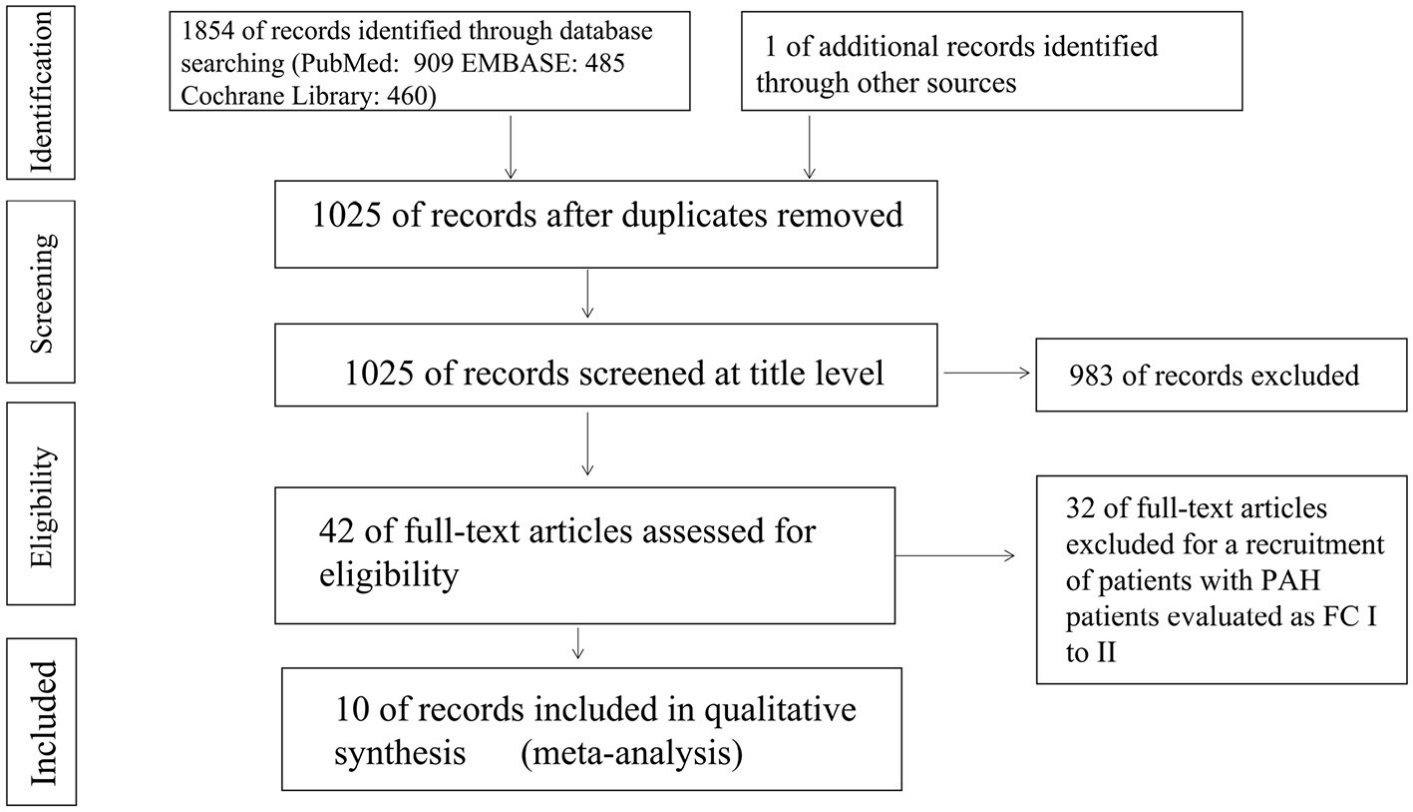
Flowchart of screening.

A total of 1,854 articles were independently retrieved by two researchers, including 909 from PubMed, 485 from EMBASE, and 460 from the Cochrane Library. After manual screening, only one eligible study was identified. After removing duplicates, 1,025 articles were selected for further consideration based on title and abstract reviews. Upon excluding 983 articles such as systematic reviews, case reports, animal or cell experiments, we proceeded to conduct a full-text reading of the remaining 42 articles. Among them, 32 studies involving PAH patients with New York Heart Association (NYHA) functional class II were excluded, resulting in the final inclusion of 10 randomized controlled trials (RCTs) (15–24). These RCTs comprised three studies on bosentan (15–17), five studies on prostacyclin analogs (18–22), and two studies on combination treatments (23, 24). A total of 311 PAH patients in the targeted drug treatment group and 242 patients in the placebo group were included in the final analysis. The screening process is summarized in Figure 1, and the baseline characteristics of the included studies are presented in Table 1. All PAH patients included in the studies had NYHA functional class III-IV at baseline.

**Table 1:** Included studies.

**References**	**Groups (No.)**	**Exposure duration (weeks)**	**IPAH /APAH (%/%)**	**Age (yrs)**	**6MWD standard**	**Hemodynamic variables**			**Types**	**FC (%) III/IV**
						**mPAP**	**PVR**	**CI(l/min/m** ^2^ **)**		
Channick et al. (15)	Bosentan (21)	12	81/19	52.2(12.2)	150-500m	54(13)	11.2(5.3)	2.4(0.7)	RCTs	100.0/0(W)
	Placebo (11)	12	91/9	47.4(14.0)		56(10)	11.8(5.4)	2.5(1.0)		100.0/0 (W)
Rubin et al. (16)	Bosentan (144)	16	71/29	48.7 (15.8)	150–450 m	55 (16)	12.7 (8.5)	2.4 (0.8)	RCTs	90.0/10.0 (W)
	Placebo (69)	16	70/30	47.2 (16.2)		53 (17)	11.0 (6.8)	2.4 (0.7)		94.0/6.0 (W)
Galie et al. (17)	Bosentan (37)	16	0/100 (ES)	37.0 (12.0)	150–450 m	77.8 (15.2)	42.8 (17.6)	NR	RCTs	100.0/0 (W)
	Placebo (17)	16	0/100 (ES)	44.2 (8.5)		72.1 (19.4)	35.9 (15.1)	NR		100.0/0 (W)
Barst et al. (18)	Epoprostenol (41)	12	100/0	40.0 (3.0)	NR	61 (2)	16 (1)	2.0 (0.1)	RC Ts	76.0/24.0 (N)
	Conventional (40)	12	100/0	40.0 (2.0)		59 (2)	16 (1)	2.1 (0.2)		73.0/28.0 (N)
Olschewski et al. (19)	Iloprost (101)	12	50.5/49.5	51.2 (13.2)	50–500 m	52.8 (11.5)	12.9 (4.9)	3.8 (1.1)	RCTs	59.4/40.6 (N)
	Placebo (102)	12	50.0/50.0	52.8 (12.0)		53.8 (14.1)	13.0 (6.2)	3.8 (0.9)		57.8/42.2 (N)
McLaughlin et al. (20)-TRIAL3	Treprostinil (17)	8	100/0	37.0 (17.0)^*^	NR	59.0 (4.0)	24.8 (2.6)	2.3 (0.2)	RCTs	96.0/4.0 (N)^*^
	Placebo (9)	8	100/0			64.0 (6.0)	24.7 (3.0)	2.4 (0.2)		
Hirematch et al. (21)	Treprostinil (30)	12	97/3	30 (12.5)	50–325 m	64.0 (3.0)	24.5 (2.34)	2.7 (0.3)	RCTs	100.0/0 (N)
	Placebo (14)	12	93/7	36 (11.3)		66.0 (6.0)	29.1 (5.84)	2.6 (0.5)		100.0/0 (N)
McLaughlin et al. (22)	Treprostinil (120)	12	56/44	55 (20–75)	200–45	NR	NR	NR	RCTs	93.3/2.5 (N)
	Placebo (120)	12	56/44	52 (18-75)	0 m					98.3/1.7 (N)
Humbert et al. (23)	Bosentan+ Epoprostenol (22)	16	77/23	45 (17)	NR	59.2 (4.0)	14.4 (1.6)	1.7 (0.1)	RCTs	73.0/27.0 (N)
	Placebo+ Epoprostenol (11)	16	91/9	47 (19)		60.9 (2.9)	17.8 (1.8)	1.7 (0.2)		77.0/23.0 (N)
Heoper et al. (24)	Iloprost+Bosentan (19)	12	100/0	48.0 (14.0)	150–425 m	54.0 (12.0)	13.5 (6.6)	2.1 (0.7)	RCTs	100.0/0 (W)
	Placebo+Bosentan (21)	12	100/0	56.0 (13.0)		59.0 (19.0)	12.9 (6.7)	2.1 (0.5)		100.0/0 (W)

No., number; ES, Eisenmenger syndrome; NR, not report; mPAP, mean pulmonary artery pressure; PVR, pulmonary vascular resistance; CI, cardiac index; RCTs, randomized controlled trials; FC, functional class; W, WHO criteria; N, NYHA criteria. ^*^The data were described for all patients in the enrolled trial.

### Quality assessment

**Figure 2:**
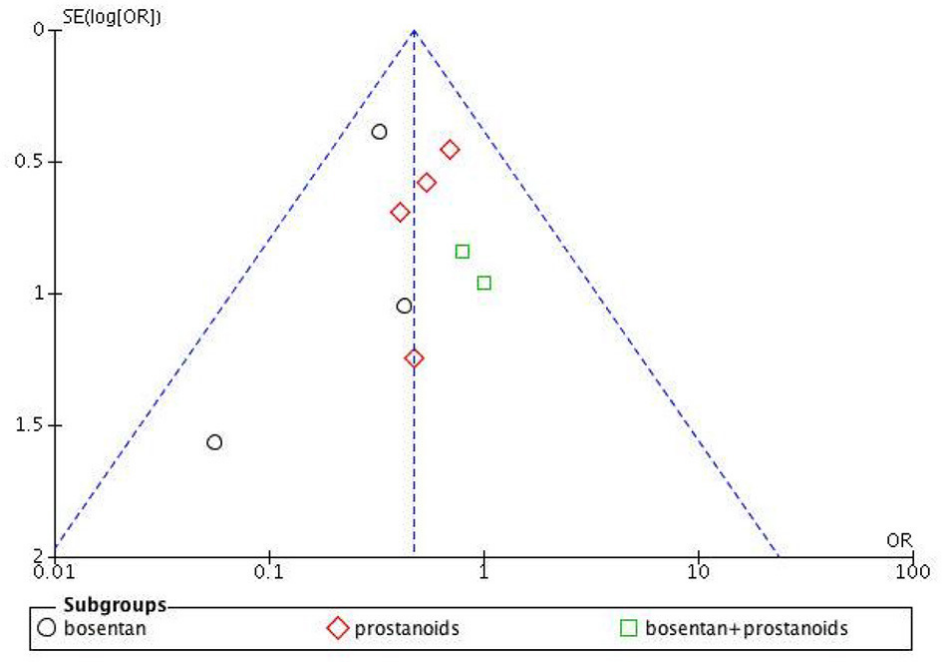
Funnel plot of clinical worsening events.

The assessment of bias risk for the studies included in our analysis is depicted in Supplementary Figures S1 and S2. The funnel plot for clinical deterioration events (Figure 2) indicates no significant bias among the studies, and both Begg's test and Egger's test for 6MWD reveal no apparent bias (Begg's test *p* = 0.087, Egger's test *p* = 0.354).

### Safety

The adverse reactions associated with targeted drugs are mostly mild to moderate, such as liver function abnormalities, headaches, and dizziness in monotherapy with bosentan, occurring at a rate of approximately 13.5%. When administered as monotherapy, prostaglandin analogs can manifest as cough or flu-like symptoms, headaches, and gastrointestinal symptoms, occurring at a rate of ~12.1%. In patients receiving combination therapy, adverse reactions including gastrointestinal symptoms, jaw pain, and flushing have been reported, with an incidence rate of 13.6%. The detection rate in the bosentan group is approximately 13.5%, and the specific details are outlined in Table 2.

**Table 2:** Meta-analysis and subgroup analysis of frequency in adverse events.

**Adverse events**	**Bosentan monotherapy**	**Prostanoids monotherapy**	**A combination**
**Any event**	22.8% (16.5%, 29.1%)	66.6% (63.0%, 70.2%)	36.8% (15.2%, 58.5%)
Headache	19.4% (6.4%, 32.5%)	36.7% (27.0%, 46.3%)	20.4% (0.0%, 47.7%)
Dizziness or vertigo	10.3% (6.1%, 15.3%)	11.9% (4.8%, 21.2%)	5.5% (0.1%, 15.5%)
Cough or influenza-like symptoms	5.6% (1.8%, 9.3%)	64.1% (58.6%, 69.6%)	15.2% (4.4%, 26.0%)
Cardiopulmonary symptoms^*^	10.1% (0.5%, 27.8%)	23.0% (5.8%, 46.1%)	5.5% (0.1%, 15.5$)
Syncope	9.0% (4.3%, 13.7%)	7.6% (2.8%, 12.3%)	-
Flushing	9.0% (4.3%, 13.7%)	19.1% (13.9%, 24.3%)	27.3% (8.7%, 45.9%)
Gastrointestinal symptoms^**^	-	24.3% (20.1%, 28.5%)	72.7% (54.1%, 91.3%)
Jaw pain	-	8.1% (2.0%, 14.2%)	59.1% (38.5%, 79.6%)
Abnormal hepatic function	57.2% (0.0%, 100.0%)	-	-
Local symptoms	-	9.0% (4.3%, 13.7%)	9.1% (0.0%, 21.1%)
Severe intensity	13.5% (12.9%, 14.1%)	12.1% (11.9%, 12.2%)	13.6% (12.6%, 14.7%)

^*^The symptoms included dyspnea, chest pain, palpitations, or bradycardia; ^**^The symptoms included nausea, vomiting, or diarrhea. The events of severe intensity included heart failure, severe syncope, respiratory failure, et al. Data in this table was displayed as an estimated percentage (95% CrIs).

**Figure 3:**
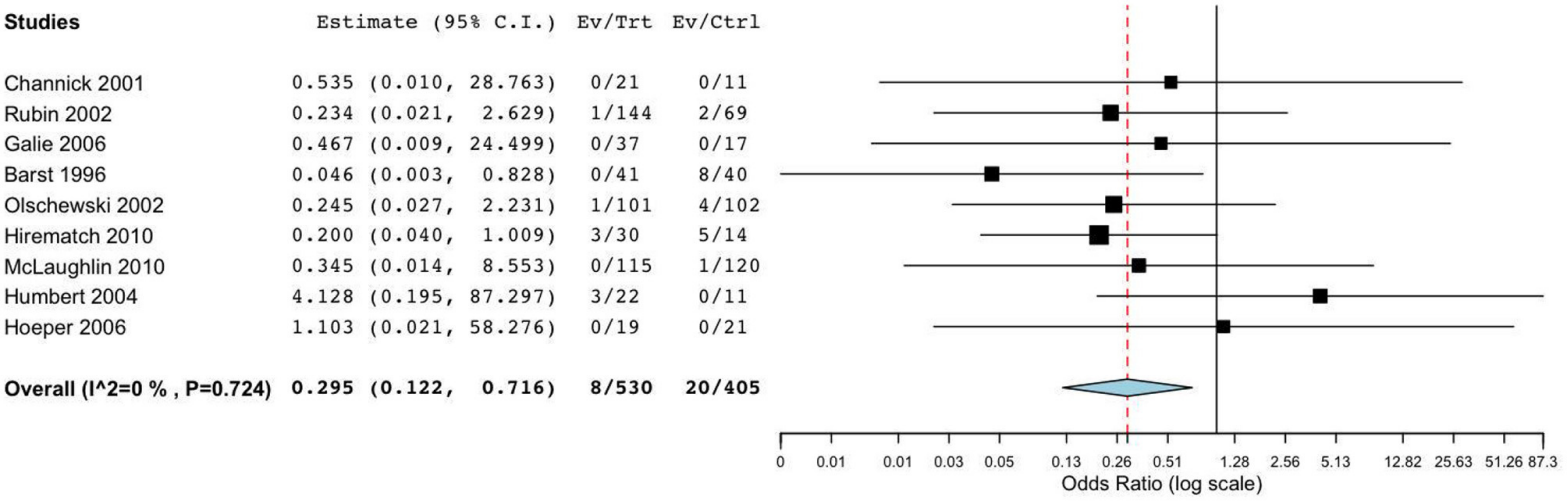
Forest plot of mortality comparing specific drugs vs. placebo.

The primary outcomes of nine RCTs (15–19, 21–23) included all-cause mortality and clinical worsening. Meta-analysis revealed that, compared to placebo, targeted drugs significantly reduced mortality (OR = 0.30, 95%CI = [0.12, 0.72], I^2^ = 0.0%, *p* = 0.007) and clinical worsening (OR = 0.48, 95%CI = [0.31, 0.73], I^2^ = 0.0%, *p* < 0.001) in patients with FC III-IV PAH. Subgroup analysis demonstrated that, compared to placebo, monotherapy with bosentan or prostaglandin analogs reduced the incidence of death or clinical worsening events in patients with III-IV PAH, and these findings were statistically significant (Figure 3). However, when compared to monotherapy with bosentan or prostaglandins, the impact of combination therapy on mortality (OR = 2.53, 95%CI [0.23, 28.4], I^2^ = 0.0%, *p* = 0.452) and clinical worsening (OR = 0.88, 95%CI [0.23, 3.03], I^2^ = 0.0%, *p* = 0.839) was not significant.

### Exercise capacity

**Figure 4:**
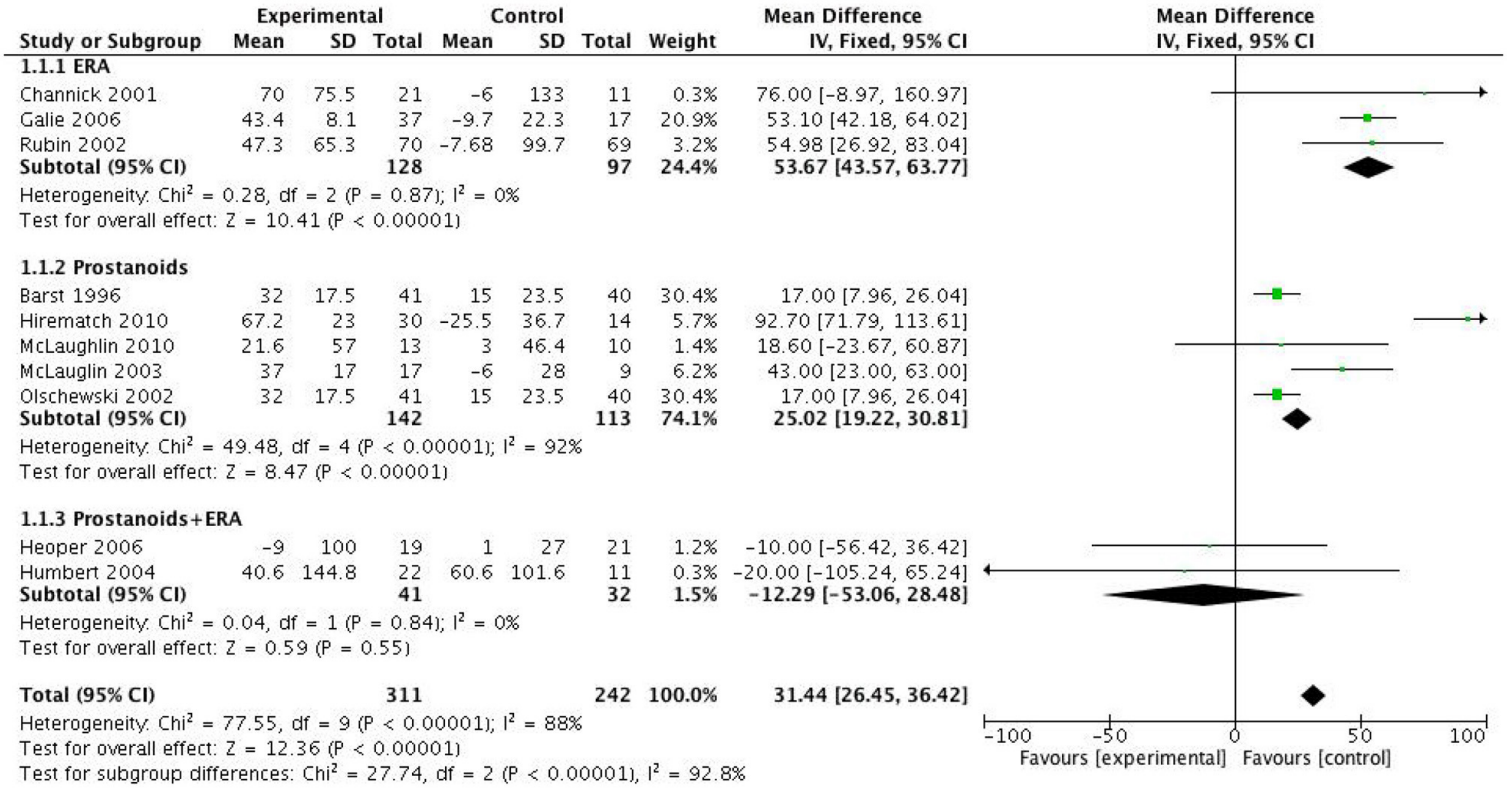
Forest plot of 6MWD comparing specific drugs vs. placebo.

Meta-analysis and subgroup analysis revealed that both monotherapy with bosentan and prostanoids significantly increased the 6MWD in PAH patients with FC III-IV, by 53.67 meters (*p* < 0.0001) and 25.02 meters (*p* < 0.0001) respectively, compared to the control group. However, studies by Heoper et al. and Humber et al. demonstrated that the combination of targeted therapies did not significantly improve 6MWD compared to monotherapy (MD = -12.29 meters, 95%CI = [-53.06, 28.48] meters, *p* = 0.55, I^2^ = 0.0%) (Figure 4).

A further analysis of cardiac function revealed that only 19.3% (95%CI = [14.9%, 23.7%]; I^2^ = 70.7%) of patients in the control group exhibited at least a one-grade improvement in NYHA/WHO cardiac function, whereas 37.2% (95%CI = [32.3%, 42.1%]; I^2^ = 71.3%) of patients treated with targeted therapies demonstrated such improvement. Among the patients treated with bosentan monotherapy, 40.3% (95%CI = [33.1%, 47.4%]; I^2^ = 0.0%) showed improved cardiac function, while 30.0% (95%CI = [22.3%, 37.6%]; I^2^ = 87.6%) of patients treated with prostanoids experienced similar benefits. Notably, the combination of targeted therapies resulted in a higher percentage of PAH patients with improved cardiac function, at 59.1% (95%CI = [38.5% to 79.6%]).

### Hemodynamics

Six RCTs (15, 17–20, 23) have evaluated hemodynamic parameters, and meta-analysis and subgroup analysis data indicated significant improvements of hemodynamics, including mPAP (Supplementary Figure S3) and PVR (Supplementary Figure S4). Actually, compared to placebo, monotherapy with bosentan (SMD = −1.07, 95% CI = [−2.08, −0.06], *p* = 0.04) or prostacyclin analogs (SMD = −1.26, 95% CI = [−2.21, −0.32], *p* = 0.009) significantly reduces PVR in patients with PAH at FC III-IV stages, suggesting substantial improvement in pulmonary vascular remodeling in this subset of patients. However, there is currently a lack of reports on the hemodynamic effects of combined targeted drug treatments.

## Discussion

Currently, the primary clinical objective in PAH treatment is to enhance patients' long-term quality of life (12, 25). Clinical guidelines typically recommend targeted drug therapies for PAH based on the WHO/NYHA Functional Class (FC) grading system (26, 27). For patients with PAH classified as FC III-IV, intravenous epoprostenol is recommended as a first-line (IA-level) treatment. While this drug significantly improves patients' exercise tolerance, clinical symptoms, and hemodynamics, some patients may experience severe local adverse effects. Although other monotherapy or combination therapies are recommended as IIb or C-level treatments, there is currently a lack of evidence supporting the clinical efficacy and safety of alternative targeted drugs in treating FC III-IV PAH (28–30).

To address this gap, our study systematically evaluated and analyzed 10 RCTs specific to this patient population, providing evidence on the efficacy and safety of targeted drug treatments for PAH. We conducted a rigorous assessment of the quality and bias of each RCT, confirming the absence of low-quality studies and no significant publication bias. Our analysis included RCTs on bosentan monotherapy, prostacyclin monotherapy (including epoprostenol, iloprost, and treprostinil), and combination therapy with bosentan and prostacyclin analogs. Treatment durations ranged from 8 to 16 weeks, and outcomes were evaluated based on exercise tolerance and FC grading.

Safety analysis via Meta-analysis revealed that targeted drug therapy significantly reduced mortality (OR = 0.30, 95%CrIs [0.12, 0.72], *p* = 0.007) and the risk of clinical worsening events (OR = 0.48, 95%CI = [0.31, 0.73], *p* < 0.001) in FC III-IV PAH patients. Compared to placebo, single-target drugs significantly improved patients' 6-Minute Walk Distance (6MWD). Furthermore, hemodynamic assessments demonstrated that monotherapy with targeted drugs significantly reduced Pulmonary Vascular Resistance (PVR) in PAH patients with FC III-IV, indicating improved lung vascular remodeling.

A comparative analysis of combined targeted therapy vs. monotherapy revealed that while the combination of endothelin pathway modulators (such as bosentan) and prostacyclin analogs did not significantly improve 6MWD in the short term, it did result in a more pronounced reduction in FC grading compared to monotherapy. Notably, there was no significant increase in the incidence of severe adverse drug reactions with combination therapy (13.6% for combination vs. 13.5% for prostacyclin analogs vs. 12.1% for bosentan). However, data on the effect of combination therapy on lung vascular function in this patient population remains limited.

According to clinical guidelines, combination therapy with two or more targeted drugs targeting the endothelin, prostacyclin, and nitric oxide pathways is recommended (12, 31). With increasing experience in combination therapy, a Meta-analysis has shown that it can reduce the risk of clinical worsening, improve exercise capacity, and promote lung vascular remodeling, although it does not significantly reduce all-cause mortality (32).

Focusing on treatment strategies for PAH patients with severe heart failure (33), our study included patients with FC III or IV PAH. The results indicate that short-term use of targeted drugs can significantly enhance exercise tolerance and alleviate lung vascular remodeling. Interestingly, we found that in PAH patients with moderate to severe heart failure, short-term combination therapy led to more improvements in cardiac function compared to monotherapy, although the increase in 6MWD was not significant. This may be attributed to the relatively short duration of treatment. Notably, one study demonstrated the long-term benefits of triple therapy with bosentan, sildenafil, and tadalafil in FC III-IV PAH patients, suggesting that early and prolonged treatment with hemodynamic monitoring should be considered for this population.

In conclusion, while monotherapy with targeted drugs can significantly improve exercise tolerance and lung vascular remodeling in FC III-IV PAH patients, combination therapy may further enhance efficacy with reasonable safety. Additionally, incorporating risk-based stratification for PAH patients should be carried out, especially for patients with heart function III-IV patients. These patients should be timely treated with targeted drug therapy and efficacy evaluation. However, long-term observation and clinical studies are needed to validate it.

## Data Availability

The original contributions presented in the study are included in the article/Supplementary material, further inquiries can be directed to the corresponding author.
