# Supplementary material for: The specific medications for pulmonary arterial hypertension at functional class III to IV: a systematic review and meta-analysis
Source: Front Med (Lausanne). 2024 Dec 12;11:1448503. doi: 10.3389/fmed.2024.1448503 (PMC11672201; doi:10.3389/fmed.2024.1448503)
Supplement: Supplementary file 1 [file Presentation_1.pdf]

The specific medications for pulmonary arterial hypertension at functional class III to IV: A systematic review and meta-analysis

Supplementary materials

4 Figures

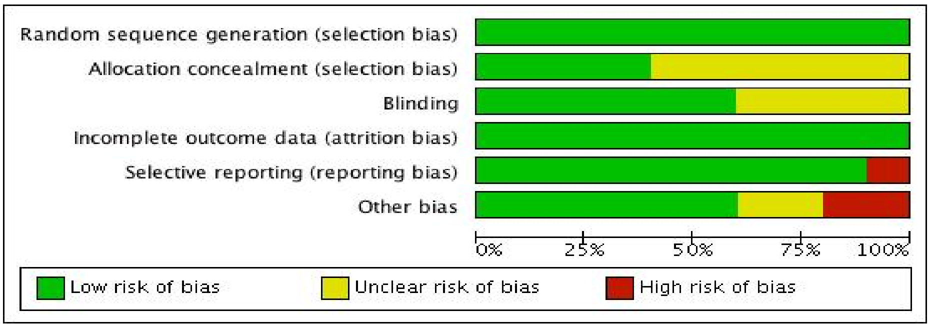

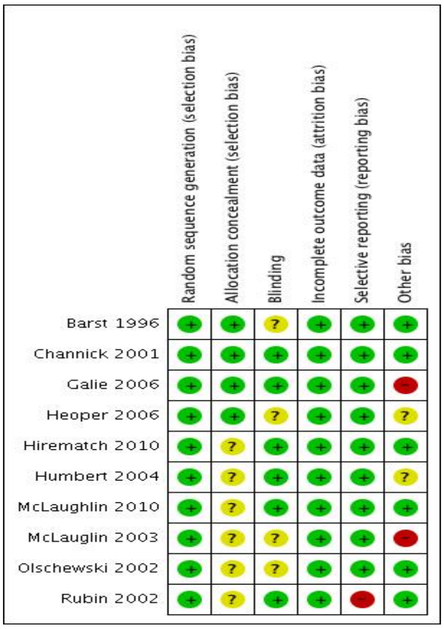

Figure S1 and S2. Risk of bias

Study or Subgroup	Mean	SD	Total	Mean	SD	Total	Weight	Mean Difference IV, Random, 95% CI
**group = ERA**								
Channick	-1.600	5.4000	20	5.100	8.8500	10	13.3%	-6.700 [-12.674, -0.726]
Galie	-5.000	9.1200	37	0.500	5.8000	17	17.2%	-5.500 [-9.530, -1.470]
**Total (95% CI)**			**57**			**27**	**30.5%**	**-5.875 [-9.216, -2.535]**
Heterogeneity: $Tau^2 = 0$; $Chi^2 = 0.11$, df = 1 (P = 0.74); $I^2 = 0\%$								
Test for overall effect: Z = -3.45 (P < 0.01)								
**group = PRO**								
Barst	-2.400	7.0400	41	-1.400	8.2200	40	18.6%	-1.000 [-4.337, 2.337]
Oleschewski	-4.600	9.3000	101	-0.200	6.9000	102	20.7%	-4.400 [-6.654, -2.146]
MacLauglin-1	0.500	3.0000	15	-1.500	1.0000	9	21.6%	2.000 [0.347, 3.653]
**Total (95% CI)**			**157**			**151**	**60.9%**	**-1.089 [-5.365, 3.186]**
Heterogeneity: $Tau^2 = 12.688$; $Chi^2 = 20.31$, df = 2 (P < 0.01); $I^2 = 90\%$								
Test for overall effect: Z = -0.50 (P = 0.62)								
**group = PRO+ERA**								
Humbert	-6.700	16.4000	22	-1.700	10.2000	11	8.5%	-5.000 [-14.127, 4.127]
Test for overall effect: Z = -1.07 (P = 0.28)								
**Total (95% CI)**			**236**			**189**	**100.0%**	**-2.929 [-6.305, 0.446]**
Heterogeneity: $Tau^2 = 13.010$; $Chi^2 = 30.40$, df = 5 (P < 0.01); $I^2 = 84\%$								
Test for overall effect: Z = -1.70 (P = 0.09)								
Test for subgroup differences: $Chi^2 = 3.03$, df = 2 (P = 0.22)								

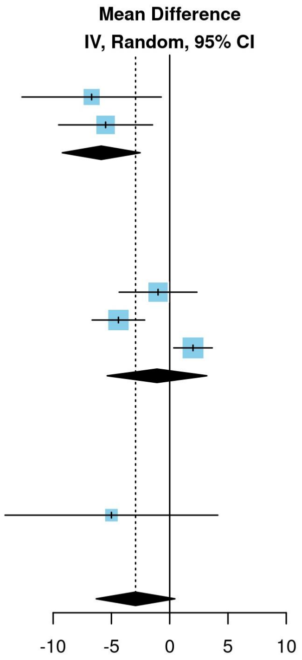

Figure S3. Forest plot of mPAP comparing specific drugs versus placebo.

Study or Subgroup	Mean	SD	Total	Mean	SD	Total	Weight	Std. Mean Difference IV, Random, 95% CI
group = ERA								
Channick	-223.000	250.4400	20	191.000	234.0000	10	14.3%	-1.642 [-2.522, -0.763]
Galie	-316.900	841.2000	37	155.100	552.5000	17	18.0%	-0.609 [-1.195, -0.022]
Total (95% CI)			57			27	32.3%	-1.071 [-2.079, -0.064]
Heterogeneity: $Tau^2 = 0.389$; $Chi^2 = 3.67$, df = 1 (P = 0.06); $I^2 = 73\%$								
Test for overall effect: Z = -2.08 (P = 0.04)								
group = PRO								
Barst	-5.500	5.1200	41	-3.900	5.0600	40	19.8%	-0.311 [-0.750, 0.127]
Olschewski	-239.000	279.0000	101	96.000	322.0000	102	21.3%	-1.107 [-1.403, -0.812]
MacLauglin-1	-4.800	1.4000	15	0.200	1.9000	9	10.4%	-3.017 [-4.260, -1.774]
Total (95% CI)			157			151	51.6%	-1.265 [-2.208, -0.322]
Heterogeneity: $Tau^2 = 0.572$; $Chi^2 = 19.85$, df = 2 (P < 0.01); $I^2 = 90\%$								
Test for overall effect: Z = -2.63 (P < 0.01)								
group = PRO+ERA								
Humbert	-564.000	555.8000	22	-376.000	489.2000	11	16.2%	-0.343 [-1.072, 0.386]
Test for overall effect: Z = -0.92 (P = 0.36)								
**Total (95% CI)**			**236**			**189**	**100.0%**	**-1.011 [-1.556, -0.467]**
Heterogeneity: $Tau^2 = 0.339$; $Chi^2 = 25.87$, df = 5 (P < 0.01); $I^2 = 81\%$								
Test for overall effect: Z = -3.64 (P < 0.01)								
Test for subgroup differences: $Chi^2 = 2.72$, df = 2 (P = 0.26)								

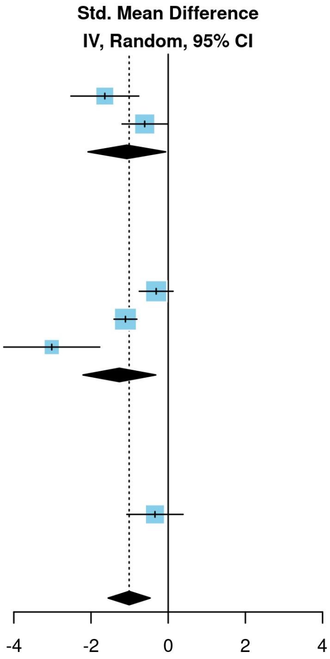

Figure S4. Forest plot of PVR comparing specific drugs versus placebo.
